# Robot-Assisted Osteotomy and Reconstruction with AR Guidance in Maxillofacial Reconstructive Surgery

**DOI:** 10.34133/cbsystems.0590

**Published:** 2026-05-22

**Authors:** Sifan Cao, Jingfan Fan, Long Shao, Qing Sun, Tao Xu, Danni Ai, Tianyu Fu, Deqiang Xiao, Hong Song, Tao Zhang, Jian Yang

**Affiliations:** ^1^ Beijing Key Laboratory for Surgical Navigation Robots with Augmented Reality, School of Optics and Photonics, Beijing Institute of Technology, Beijing 100081, China.; ^2^ Zhengzhou Research Institute, Beijing Institute of Technology, Zhengzhou 450003, China.; ^3^ Department of Stomatology, Peking Union Medical College Hospital, Chinese Academy of Medical Sciences and Peking Union Medical College, Beijing 100730, China.; ^4^ Beijing Key Laboratory for Surgical Navigation Robots with Augmented Reality, School of Medical Science and Engineering, Beijing Institute of Technology, Beijing 100081, China.; ^5^ Beijing Key Laboratory for Surgical Navigation Robots with Augmented Reality, School of Computer Science and Technology, Beijing Institute of Technology, Beijing 100081, China.

## Abstract

The treatment of maxillofacial tumors requires coordinated resection and reconstruction. Conventional template-guided open osteotomy techniques may increase the risk of tissue injury. Additionally, the accuracy of titanium plate-based reconstruction is limited by the lack of standardized positional criteria, thereby potentially affecting alignment consistency. To address these challenges, we propose a maxillofacial tumor treatment system termed RAMRS, comprising 2 modules: robot-assisted osteotomy and augmented reality-guided reconstruction (ARR). Within therobot-assisted osteotomy module, a hand–eye calibration framework is used to support robotic arm positioning, while preoperative CT-to-patient spatial alignment is achieved through an optical probe-mediated registration method, supporting robot-assisted osteotomy according to the preoperative plan. The ARR module incorporates a rotating-caliper-based calibration compensation framework to reduce marker registration errors caused by manual instability or fiducial marker deformation. Subsequently, quick-response markers are used to achieve spatial alignment between the preoperative 3-dimensional model and the intraoperative defective mandible, and the virtual mandibular model is projected onto a display visible to the surgeon, thereby providing intuitive intraoperative guidance. Validation experiments were conducted on cadaveric and ex vivo specimens. The results demonstrated favorable accuracy in osteotomy and reconstruction alignment in the evaluated experiments, while the ARR module achieved low 2-dimensional fusion error for augmented reality visualization. These findings support the feasibility of the proposed workflow in preclinical settings.

## Introduction

Maxillofacial tumor resection often results in substantial tissue defects. The fibular flap is widely used for mandibular reconstruction due to its sufficient bone stock and limited donor-site morbidity [1]. However, adapting a linear fibula to the mandible’s complex curved contour requires high geometric accuracy in osteotomy planning and segment assembly. Accurate spatial positioning and error control are therefore critical to achieving favorable postoperative aesthetics and functional recovery. Early reconstruction relied on manual determination of osteotomy parameters and freehand shaping based on preoperative imaging [2], which is prone to deviations in spatial positioning and symmetry [3,4]. Digital workflows combining virtual surgical planning and patient-specific guides have improved efficiency [5,6], yet their performance is often limited by imperfect fitting on smooth bony surfaces. This may necessitate wider soft-tissue dissection for stabilization, increasing surgical trauma [7,8]. Moreover, the lack of rigid spatial constraints during the critical repositioning phase often requires iterative manual adjustments, which can lead to complications such as malocclusion or condylar displacement.

Robotic-assisted surgery (RAS) has shown potential for improving execution consistency and motion control in surgical tasks [9]. Despite recent robotic schemes based on path planning or multisource tracking [10,11], achieving verifiable real-time tracking error control under intraoperative uncertainties, such as patient micromovement, remains challenging. Han et al. [12] applied RAS to mandibular angle osteotomy, but accuracy was constrained by reliance on manual markers and preset points. Li et al. [13] combined preoperative planning with robotic navigation, yet validation remained largely preclinical. Hu et al. [14] proposed sensor-aware hybrid control for force-controlled cutting, but control over key dimensions such as osteotomy depth was insufficient. Overall, robust real-time spatial registration between instruments and anatomical targets remains a major hurdle due to occlusion, micromotion, and positioning uncertainties.

To improve spatial awareness during reconstruction, augmented reality (AR) has been investigated as a means of overlaying preoperative plans onto the surgical field and providing intuitive visual guidance for intraoperative alignment and segment repositioning [15]. For craniomaxillofacial AR guidance, marker-based tracking has been commonly used because it provides an explicit registration chain that is easier to calibrate and evaluate quantitatively [16]. Recent research has also shifted from qualitative visual demonstrations toward quantitative evaluation with controllable and traceable errors. Brunzini et al. [17] proposed a hybrid reality scheme for maxillofacial osteotomy, yet localization bias between markers and plans can still accumulate across multiple stages. García-Mato et al. [18] explored AR-guided navigation with electromagnetic tracking in craniofacial surgery. However, their practical performance may still be affected by environmental disturbance, calibration burden, and workflow complexity. In practice, effective marker-based AR depends on maintaining a stable marker–anatomy relationship while minimizing cumulative calibration errors.

Given the limitations of single-modality technologies, integrating AR and robotics has gained increasing attention [19]. Early explorations of combining AR and RAS in craniofacial surgery demonstrated the feasibility of robot-supported localization tasks [20]. More recent studies have explored AR-integrated or robot-assisted strategies for craniofacial osteotomy and reconstruction. For example, Zhou et al. [21] incorporated AR visualization into robot-assisted mandibular procedures, while Han et al. [12] investigated optical-tracking-based collaborative robotic osteotomy. Related efforts have also examined optical-tracking-supported planning and guidance [22] and AR-based visualization of osteotomy lines [17]. Nevertheless, existing studies often focus on isolated stages of the workflow or rely on separate guidance and execution modules. This makes it difficult to maintain continuous pose constraints and to quantitatively evaluate the full osteotomy-to-reconstruction process within a unified framework.

To address these issues, this study proposes the robot-assisted and AR-guided maxillofacial reconstruction system (RAMRS) to provide continuous constraints and feedback through a unified workflow. During the robot-assisted osteotomy (RAO) phase, a collaborative robotic arm enforces rigid motion constraints, enabling guide-free precision cutting while minimizing tissue exposure. During the AR-guided reconstruction (ARR) phase, the system dynamically aligns preoperative plans with intraoperative anatomy and provides real-time pose references to improve repositioning consistency. To assess translational potential, we conducted systematic validation ranging from phantoms to cadaveric specimens and quantified multiple deviations to evaluate both geometric precision and procedural efficiency.

The main contributions of this work are as follows:

1. We propose a novel maxillofacial reconstruction system integrating robot-assisted surgery and AR. The system enables rapid calibration, precise osteotomy, and repositioning guidance, thereby improving reconstruction accuracy.
2. We develop an AR-guided fibular segment positioning method that provides surgeons with intuitive visual guidance for maxillofacial reconstruction.
3. We introduce a rotating-calipers-based compensation framework for point–plane registration to improve the accuracy of AR fusion.

## Materials and Methods

**Fig. 1.**
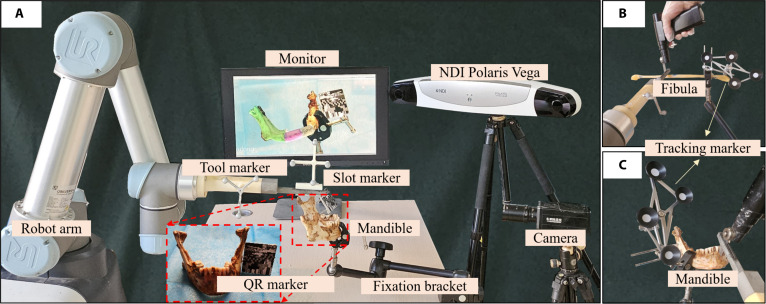
System hardware setup. (A) All devices of the robot-assisted and augmented reality-guided maxillofacial reconstruction system (RAMRS). (B) Fibula osteotomy scene. (C) Mandible osteotomy scene.

Fig. 1A displays several components used during the osteotomy and reconstruction process, including an NDI Polaris Vega optical tracking system with a frame rate of 60 Hz (NDI, Northern Digital Inc.), 2 optical markers, a quick-response (QR) marker, a robotic arm (UR5, Odense, Denmark), a fixation bracket (KCP-101, Israel), a computer with a display, and an industrial camera (SY8031, Shenzhen, China). Specifically, the tool marker and slot marker are used to determine the end-effector pose of the robotic arm and the position of the osteotomy slot. After hand–eye calibration and target alignment, RAMRS guides the robotic arm to the planned osteotomy area. The industrial camera continuously tracks the QR marker, and once it is aligned with the coordinate system of the defective mandible, the monitor displays both the real-world view and the AR overlay of the preoperative plan to provide visual guidance for the surgeon. Fig. 1B and C illustrate the setup for bone tracking and osteotomy. Once rigidly affixed to bone using self-tapping screws, the tracking markers enable real-time motion tracking of osseous structures. After the robotic arm positions the osteotomy slot in the planned area, the surgeon uses a saw blade to perform the osteotomy under slot guidance.

**Fig. 2.**
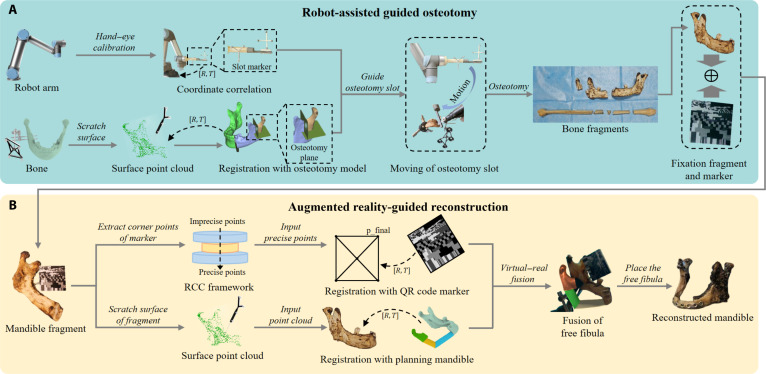
Flowchart of the robot-assisted and augmented reality-guided maxillofacial reconstruction system (RAMRS). (A) Intraoperative robot-assisted osteotomy process. (B) Intraoperative augmented reality-guided reconstruction process.

The workflow of the RAMRS system is illustrated in Fig. 2. After obtaining the patient’s CT data, the surgeon defines the osteotomy planes of the fibula and mandible during preoperative planning and generates the corresponding planning models. Fibular segments are arranged according to the mandibular defect morphology to construct the planned mandibular reconstruction model. Intraoperatively, as shown in Fig. 2A, a tracking marker is implanted into the fibula or mandible to provide a stable reference. After exposing the bony structures, an optical probe is used to acquire the bone-surface point cloud (SPC) and register the preoperative plan to the real anatomy, and the system outputs the registration error to assess registration accuracy. After registration and calibration, the robotic arm automatically aligns the osteotomy slot with the preplanned trajectory, and the surgeon performs bone resection using a medical bone saw under slot-imposed angular constraints and real-time tracking. In the reconstruction phase (Fig. 2B), a QR marker is fixed to the defective mandible via a guide plate. An optical probe samples the QR corners, which are refined by the rotating-calipers-based compensation (RCC) framework to achieve precise marker-to-mandible alignment. Once the QR marker and the mandible are coregistered in a unified coordinate system, the camera captures the mandibular surface geometry and the QR-marker pose information, and the preoperative mandible model is overlaid onto the video stream for real-time AR display. This provides visual guidance for pose adjustment and enables accurate positioning and reduction of the fibular segments before titanium-plate fixation.

### Preoperative planning of osteotomy and reconstruction

CT scans of mandibular and fibular structures were segmented using ProPlan CMF (Materialise HQ, Belgium), generating bone models. Considering the tomographic acquisition method of CT scanners, Laplacian smoothing [23] was applied to the initially segmented models to achieve smoother edges of the bone models, thereby better approximating the morphology of real bone specimens. Surgeons designed osteotomy planes according to clinical osteotomy criteria. Given that maxillofacial tumors typically exhibit bony invasion, the osteotomy planes incorporated a redundant design to balance minimal surgical trauma with complete lesion removal. Subsequently, fibular segments were morphologically matched and aligned with the mandibular defect to establish the reconstructed mandibular model.

### Robot-assisted osteotomy

Robot-assisted surgery provided surgeons with enhanced operational precision and mitigated fatigue-related human errors during prolonged procedures. Compared to existing maxillofacial osteotomy methods [2,7,16,24–26], the lack of positional guidance and angular constraints was consistently a key challenge hindering surgeons from performing precise operations. Therefore, we developed an optical tracking-guided intraoperative navigation scheme. This scheme enables system-to-patient spatial localization and real-time patient tracking under intraoperative micro-motion, thereby supporting precise osteotomy in the RAO module.

#### Robotic arm calibration

**Fig. 3.**
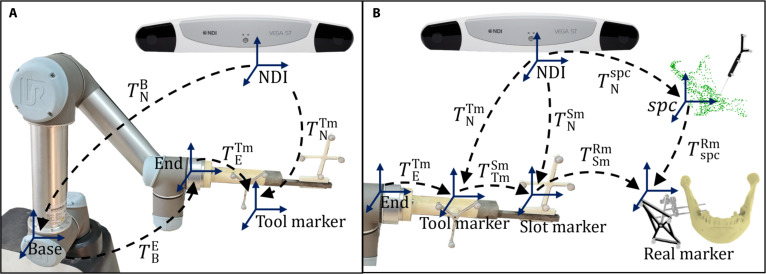
Intraoperative pose transformation relationships for robotic-assisted surgery (RAS)-assisted osteotomy. (A) Schematic of robotic arm hand–eye calibration. (B) Schematic of spatial pose alignment between the robotic arm and the patient in the physical space.

The purpose of extrinsic calibration was to determine the relative pose between multiple components [27]. In the RAO module, since the pose of the robotic arm end-effector relative to the tool marker was unknown, hand–eye calibration algorithms [28,29] were used to compute the transformation matrix between them. The osteotomy jig was mounted at the end-effector of the robotic manipulator. This jig integrated a self-localizing optical marker, an osteotomy slot for bone saw insertion, and a slot marker. As shown in Fig. 3A, the tool marker, the manipulator base, and the end-effector satisfied the following constraint relation regardless of the manipulator pose:

$$T_{N}^{B}\cdot T_{B}^{E}\cdot T_{E}^{\mathrm{Tm}}=T_{N}^{\mathrm{Tm}}$$ (1)

$T_{N}^{B}$, $T_{B}^{E}$, and $T_{N}^{\mathrm{Tm}}$ represented the positional transformation matrices from the NDI coordinate system to the robotic arm base, from the robotic arm base to the end-effector, and from the NDI coordinate system to the tool marker, respectively. The matrix $T_{B}^{E}$ was obtained from the robotic arm controller, whereas $T_{N}^{\mathrm{Tm}}$ was measured by the NDI optical tracking system. Hand–eye calibration can be formulated as a set of equations in the form of AX=YB, with the goal of estimating the transformation between the tool marker and the robotic arm end-effector. By moving the robotic arm end-effector to multiple poses, the corresponding transformation matrices $T_{B}^{E}$ and $T_{N}^{\mathrm{Tm}}$ were recorded. As $T_{E}^{\mathrm{Tm}}$ and $T_{N}^{B}$ remained constant during the calibration process, the following equation held for each pose *i*:

$${T_{B}^{E}}^{i}\cdot T_{E}^{\mathrm{Tm}}={\left(T_{N}^{B}\right)}^{-1}\cdot {T_{N}^{\mathrm{Tm}}}^{i}$$ (2)

where ${T_{B}^{E}}^{i}$ and ${T_{N}^{\mathrm{Tm}}}^{i}$ represent the transformation matrices measured at the ith pose (i=1,2,…,n). Accordingly, Eq. 2 can be rewritten in the form $A_{i}X=YB_{i}$, where $A_{i}={T_{B}^{E}}^{i}$, $B_{i}={T_{\text{N}}^{\text{Tm}}}^{i}$, $X=T_{E}^{\mathrm{Tm}}$, and $Y={\left(T_{N}^{B}\right)}^{-1}$. Shah’s method [30] was then employed to solve the resulting set of equations from multiple poses, thereby determining $T_{E}^{\mathrm{Tm}}$ and $T_{N}^{B}$.

#### Osteotomy area localization and bone tracking

The key to achieving accurate navigation in RAMRS was to establish the pose transformation between the robotic osteotomy slot and the preoperatively planned osteotomy plane. Therefore, we adopted a detachable slot-marker design that was rigidly coupled to the slot to represent its spatial pose, with its pose in the robotic arm end-effector coordinate system denoted as $T_{E}^{\mathrm{Sm}}$, while still allowing intraoperative removal when needed. In addition, since $T_{E}^{\mathrm{Tm}}$ had been obtained in the previous section, we could further derive:

$$T_{E}^{\mathrm{Sm}}=T_{E}^{\mathrm{Tm}}\cdot T_{\mathrm{Tm}}^{\mathrm{Sm}}$$ (3)

where $T_{\mathrm{Tm}}^{\mathrm{Sm}}$ denoted the pose transformation from the tool marker to the slot marker, which could be computed from the optical tracking system real-time measurements of both markers expressed in the same coordinate frame:

$$T_{\mathrm{Tm}}^{\mathrm{Sm}}={\left(T_{N}^{\mathrm{Tm}}\right)}^{-1}\cdot T_{N}^{\mathrm{Sm}}$$ (4)

Registration enabled estimation of the pose relationship between the preoperative plan and the intraoperative patient [31,32]. Using the mandible as an example, the same procedure was applied to the fibula. We employed a registration method from the tip of the optical probe to the mandibular surface [33,34] to align the preoperative osteotomy model with the real mandible. During tracing, the tip of the optical probe had to maintain full contact with the bone to reduce human-induced errors. The mandible was stabilized using the fixation bracket, and the optical probe tip was used to trace the bone surface. Probe-tip coordinates were obtained through stereo matching, as shown in Fig. 3B. In clinical practice, the exposure range of the fibula should be maintained at a distance of 6 to 8 cm from both the proximal and distal joints, resulting in an exposed segment of 10 to 15 cm. Within this range, approximately 300 to 400 points were acquired using the optical probe. For mandibular registration, given the variability in surgical approaches and skin-flap retraction ranges, we collected 400 to 500 surface points in our experiments. The SPC represented the patient’s anatomical geometry, and we defined the SPC as the coordinate system of the real patient. During registration, the Super4PCS method [35] was used to estimate the transformation between the real patient point cloud and the preoperative planning model. Based on this result, we obtained the transformation matrix $T_{\mathrm{SPC}}^{\mathrm{Rm}}$ from the real patient point cloud to the preoperative planning model. Moving the robotic arm to a specified osteotomy surface was essentially equivalent to obtaining the positional transformation matrix $T_{\mathrm{Sm}}^{\mathrm{SPC}}$. As shown in Fig. 3B, in the NDI coordinate system, the robotic arm and the patient space always satisfy the following equation:

$$T_{N}^{\mathrm{Sm}}\cdot T_{\mathrm{Sm}}^{\mathrm{Rm}}=T_{N}^{\mathrm{SPC}}\cdot T_{\mathrm{SPC}}^{\mathrm{Rm}}$$ (5)

$T_{N}^{\mathrm{SPC}}$ represents the pose of the real patient point cloud relative to the NDI coordinate system, which was obtained from the aforementioned point-tracing process. Thus, the transformation matrix $T_{\mathrm{Sm}}^{\mathrm{SPC}}$ could be obtained as:

$$T_{\mathrm{Sm}}^{\mathrm{SPC}}={\left(T_{N}^{\mathrm{Sm}}\right)}^{-1}\cdot T_{N}^{\mathrm{SPC}}$$ (6)

Afterward, the system directed the robotic arm to the specified osteotomy area. The surgeon removed the slot marker and inserted the medical bone saw into the slot, thereby ensuring precise osteotomy angles within the slot-width constraint. Moreover, slight patient motion during surgery may invalidate the registration. To address this issue, an optical marker was fixed on the portion to be osteotomized. If intraoperative patient motion occurred, the patient-space pose at time t was denoted as $T_{N}^{\mathrm{SPC}}\left(t\right)$. During osteotomy, the poses at each instant satisfied the following relationship:

$$T_{N}^{\mathrm{Sm}}\left(t\right)\cdot T_{\mathrm{Sm}}^{\mathrm{SPC}}=T_{N}^{\mathrm{SPC}}\left(t\right)$$ (7)

where $T_{N}^{\mathrm{Sm}}\left(t\right)$ represents the transformation matrix of the slot marker in the NDI coordinate system at time t. Both $T_{N}^{\mathrm{Sm}}\left(t\right)$ and $T_{N}^{\mathrm{SPC}}\left(t\right)$ were obtained from the NDI optical tracking system. Accordingly, the real-time pose of the osteotomy slot relative to the patient space could be determined.

### AR-guided defective mandible reconstruction

AR offers surgeons visual guidance for apparent alignment in the visual field. Marker-based registration methods typically employ QR markers as tracking fiducials [36,37]. However, most existing studies remain at the stage of validating the feasibility of fusion and pay limited attention to accuracy optimization. As a result, current QR-based fusion methods are highly sensitive to the spatial pose alignment between physical QR markers and virtual models. If the initial pose is inaccurate, it can easily degrade the quality of the subsequent fusion. Therefore, before fusing free fibular segments with the QR marker, we applied a rotating-calipers-based calibration method to refine the QR marker and model pose.

#### RCC method and marker fusion

**Fig. 4.**
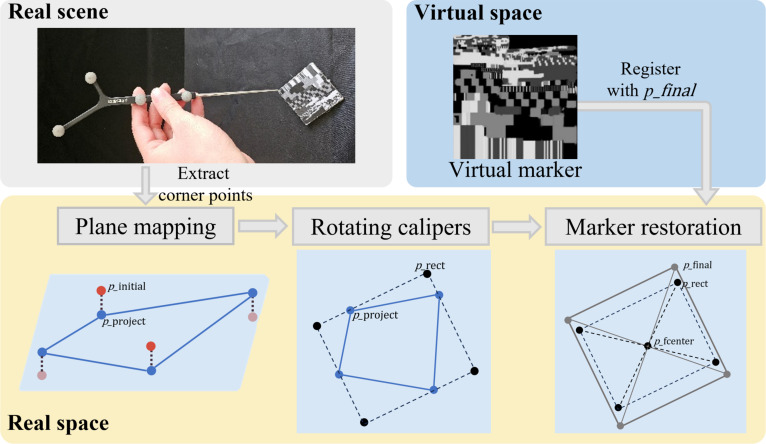
Quick-response (QR) marker registration process based on the proposed rotating-calipers-based compensation (RCC) framework.

To minimize surgical incisions and reduce adhesion between instruments and skin, tracking tools placed intraorally often use long, slender shanks. Ideally, the 4 corner points sampled by the optical probe from a QR marker lie on a common plane and form a perfect square. In practice, however, clinical complexity introduces deviations: The long lever arm can deform slightly, and hand tremor is difficult to suppress, leading to noncoplanarity and point misalignment. To mitigate these effects, we proposed a RCC method for point-plane registration, as illustrated in Fig. 4.

The RCC framework comprises 3 layers. The first layer is plane mapping. Using the probe-based picking procedure in [38], we acquired the 3-dimensional (3D) coordinates of the 4 QR corners $p_{i}^{\text{init}}={\left[x_{i},\;y_{i},\;z_{i}\right]}^{⊤},\;i\in \left\{1,\ldots ,4\right\}$. Let $\bar{p}=\frac{1}{4}\sum_{i}p_{i}^{\text{init}}$ be the centroid and $C=\frac{1}{4}\sum_{i}\left(p_{i}^{\text{init}}-\bar{p}\right){\left(p_{i}^{\text{init}}-\bar{p}\right)}^{⊤}$ the 3×3 covariance. We estimated the unit normal $n={\left[a,\;b,\;c\right]}^{⊤}$ as the eigenvector of C associated with the smallest eigenvalue (equivalently, the right singular vector with smallest singular value) and set the plane offset $d=-n^{⊤}\bar{p}$ (plane equation $n^{⊤}x+d=0$). Each point was then orthogonally projected onto the fitted plane:

$$p_{i}^{\text{proj}}=p_{i}^{\text{init}}-\frac{n^{⊤}p_{i}^{\text{init}}+d}{∥n{∥}_{2}^{2}}\;n$$ (8)

This projection enforces coplanarity and stabilizes the metric frame for subsequent steps.

The second layer is rotating calipers. Let $P_{\text{proj}}={\left\{p_{i}^{\text{proj}}\right\}}_{i=1}^{4}$ be the projected points, forming a convex quadrilateral. We sought a consistent vertex order and orientation by finding the minimum-area enclosing rectangle (MBR) over all in-plane rotations θ:

$$\theta^{⋆}=\arg\;\min_{\theta }\;\text{area}\;\left(\mathrm{MBR}\left(P_{\text{proj}},\;\theta \right)\right)$$ (9)

The rotating-calipers procedure returned a rectangle and an ordered vertex sequence $P_{\text{rect}}=\left[p_{r1,\;}p_{r2,\;}p_{r3,\;}p_{r4}\right]$ , which resolves point permutations and ensures a consistent clockwise ordering.

The third layer is marker restoration. Given the nominal QR side length s=50mm, we define a square template centered at:

$$p^{\text{center}}=\text{mean}\left(P_{\text{rect}}\right)$$ (10)

and align the template’s principal axes with those of the rectangle obtained in the second layer. By enforcing the side-length constraint, we obtain the corrected set of corner points $P_{\text{final}}\in {\mathbb{R}}^{4\times 3}$, which provides metrically consistent 3D coordinates for the QR corners in real space.

Finally, we aligned the virtual QR to $P_{\text{final}}$, enabling precise marker fusion and reliable projection of the planning mandible onto the defect site.

#### Free fibula placement and defective mandibular reconstruction

The SPC of the defective mandible (dm) was obtained. Subsequently, the preoperative planning mandible model was aligned with the SPC using the Super4PCS algorithm to obtain the spatial orientation of the defective mandible with respect to the NDI coordinate system, denoted as $T_{N}^{\mathrm{dm}}$. The positional relationship between the defective mandible and the QR marker, denoted as $T_{\mathrm{dm}}^{\mathrm{QR}}$, satisfied the following equation:

$$T_{\mathrm{dm}}^{\mathrm{QR}}={\left(T_{N}^{\mathrm{dm}}\right)}^{-1}\cdot T_{N}^{\mathrm{QR}}$$ (11)

**Fig. 5.**
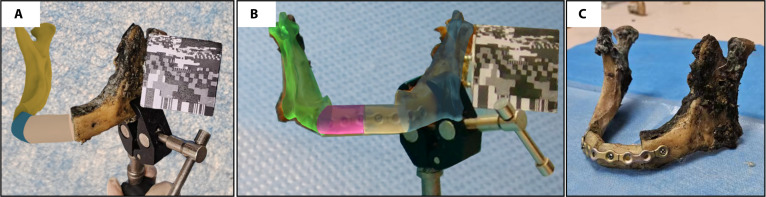
Placement of free fibula. (A) Planning mandible projects to the defect mandible. (B) Comparison with the projection after reconstruction of defect mandible. (C) The shape of the reconstructed mandible.

The preoperatively planned mandible restoration was superimposed onto a real-time video using the video perspective reality fusion display method. In Fig. 5A, the fusion scene depicted the absence of fixation for free bone segments. Utilizing various colors to represent multiple bone segments, the surgeon could extract information about the relative placement angles and edge contours between segments from the fused scene. AR technology was employed to project the placement guidance onto the truncated mandible. After the surgeon placed the bone segment in the designated position guided by the projection, a titanium plate was secured at the junction between the bone segments. Subsequently, the bone segment was drilled and fastened with titanium nails. Fig. 5B illustrates the achieved AR fusion effect after placing and fixing all free bone segments by the contours presented in the fusion scene. Fig. 5C displays the physical reconstruction of the mandible.

## Experiments and Results

### Experimental setup

The RAMRS was validated for registration and tracking errors using 25 fibula specimens and 25 mandible specimens. Osteotomy and reconstruction errors were assessed on the same specimens. To approximate a clinical setting, we additionally performed cadaveric experiments using 8 cadaver legs and 8 cadaver heads to evaluate system performance and efficiency. All instruments were sterilized before experiments. The testing team comprised 2 specialized surgeons from Peking Union Medical College Hospital and 3 engineers from our team. The study was approved by the institutional review board (Approval No.: JS-2383) and included CT scans before and after experiments (Siemens; slice thickness, 1.25 mm; 512 × 512 × 500). To reduce measurement noise, we repeated each measurement 5 times on the same specimen under the same workflow, and the average was used as the final value. In method comparisons, we used a paired design. Specifically, each method was applied to the same specimen under identical input conditions, and the resulting outputs were compared. This helped reduce the influence of interspecimen variation and experimental condition differences on the comparison results. Error measurement and statistical analysis were both conducted under single-blind conditions to reduce subjective bias. Statistical analyses were performed in SPSS, and all tests were 2-sided. For literature-comparison tables, unreported metrics were left blank, and studies reporting only means without SDs were noted. No cross-study hypothesis testing was performed because raw data were unavailable and experimental conditions were not strictly matched.

### Experiments on skeletal specimens: Osteotomy

The precision of RAO procedures may be compromised by inaccuracies in hand–eye calibration of the manipulator system. The calibration process aims to establish the critical transformation matrix through coordinate registration. However, inherent limitations including optical tracking system uncertainties and the UR5 robotic arm’s systemic errors collectively contribute to multisource interference within the RAMRS framework. Through analysis of 10 independent calibration datasets, the calibration error can be mathematically described as a pose transformation matrix encompassing rotational discrepancy ($R\in \mathrm{SO}\left(3\right)$) and translational deviation ($t\in {\mathbb{R}}^{3}$). Quantitative evaluation methods employ euler angle decomposition for rotational error analysis (providing a geometrically intuitive measure of composite rotation matrices) combined with Euclidean distance metrics for translational errors. Our experimental results demonstrate a mean angular deviation of $0.35^{\circ }\pm 0.17^{\circ }$ (mean ± SD) in rotational parameters and 1.548 mm ± 0.820 mm (mean ± SD) positional variance in translational components. Notably, the absence of universally accepted calibration standards necessitates careful interpretation of these values; they represent relative consistency metrics rather than absolute accuracy measurements, reflecting the compounded effects of multisource data integration within the surgical system.

#### CT-patient registration error

We employ the target registration error (TRE) for quantitative analysis of point-cloud registration. In this study, the TRE at a point is defined as the Euclidean distance between its measured 3D position and the corresponding position predicted after registering the CT model. The average TRE over all recorded test points is

$$V_{\mathrm{TRE}}=\frac{1}{n}\sum_{i=1}^{n}\mathrm{dis}\;\left(P_{i}^{\text{meas}},\;P_{i}^{\mathrm{CT}}\right)$$ (12)

where dis(A,B) denotes the 3D Euclidean distance between *A* and *B*, *n* is the number of test points, $P_{i}^{\text{meas}}$ is the measured coordinate on the bone surface, and $P_{i}^{\mathrm{CT}}$ is the corresponding coordinate after CT-to-surface registration.

In the registration-accuracy experiments for the mandible and fibula, 5 feature points are selected per case to compute a case-level error. For the mandible, feature points are placed near distinct anatomical junctions in the condylar region, the gonial region, and the dentition. For the fibula, which has a smoother surface, points are chosen at prominent anatomical transitions such as the intercondylar eminence, the angular region of the medial condyle, and along the interosseous margin. Exact locations adapt to specimen morphology. As summarized in Table 1, the average registration error for both bones remains submillimetric, indicating that the RAMRS registration module provides accuracy suitable for subsequent robot-guided localization.

**Table 1.** Registration error of mandible and fibula

	Mandible		Fibula	
	Mean ± SD/mm	Range/mm	Mean ± SD/mm	Range/mm
Registration error	0.58 ± 0.16	0.31–0.91	0.50 ± 0.28	0.22–0.98

#### Multiangle positioning error

**Fig. 6.**
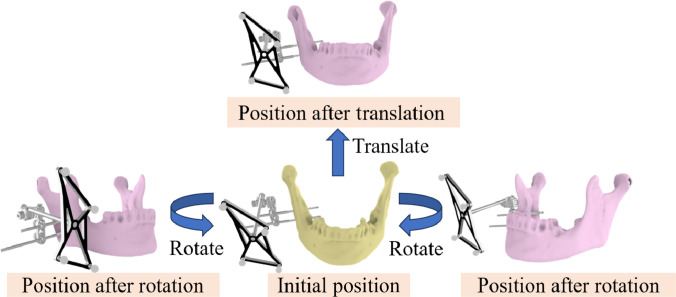
Movement diagram of the mandible embedded with a tracking marker.

Fig. 6 illustrates the multiangle positioning-error measurement using the mandible as an example. The mandible with a tracking marker is placed at K distinct poses (K=3), each exhibiting different translations and rotations with respect to the initial pose. For each pose, we compute the Euclidean distance between the measured 3D coordinates of the selected feature points and their corresponding coordinates estimated by the system. The overall tracking accuracy is reported as the root mean square error aggregated over all points and poses:

$$V_{\text{Track}}=\sqrt{\frac{1}{M}\sum_{j=1}^{K}\sum_{i=1}^{n}{\left\|P_{i,j}^{\text{meas}}-P_{i,j}^{\mathrm{sys}}\right\|}_{2}^{2}}$$ (13)

where $P_{i,j}^{\text{meas}}$ and $P_{i,j}^{\mathrm{sys}}$ denote the measured and system-estimated coordinates, respectively, for the i-th feature point at the j-th pose; n is the number of feature points per pose and M=Kn is the total number of point-pose pairs.

As summarized in Table 2, the mean multiangle tracking error remained below 1 mm, suggesting that the RAMRS tracking module can mitigate pose-induced disturbances and reduce the risk of registration failure caused by intraoperative patient position changes.

**Table 2.** Multiangle positioning error of mandible and fibula

	Mandible		Fibula	
	Mean ± SD /mm	Range (Min–Max) /mm	Mean ± SD /mm	Range (Min–Max) /mm
Positioning error	0.64 ± 0.29	0.41–0.99	0.71 ± 0.25	0.47–1.19

#### Osteotomy error

Osteotomy precision was evaluated using 2 parameters, namely, (a) the angular deviation between the resection plane and the fibular prong reference surface and (b) the Euclidean displacement of cross-sectional centroids at osteotomy sites, which quantify directional alignment and positional precision, respectively. Table 3 summarizes fibula osteotomy errors for the RAMRS system and 5 public baselines. RAMRS attained the lowest mean angular error among the reported methods and competitive centroid-distance errors. Relative to assisted computer modeling and prototyping method, the contour registration-based augmented reality system (CRAR), and combined osteotomy and reconstruction pre-shaped plate position (CORPPP), the angular deviation of RAMRS was lower by approximately 9.1%, 41.8%, and 22.4%, respectively. For centroid-distance error, RAMRS reduced the error by approximately 31.2% relative to CRAR, but it was approximately 4.1% higher than CORPPP. Its performance was close to the clinical guideline. However, the centroid-distance error remained higher than that of AR-guided osteotomy of the fibula method (AROF) with visual overlay. Overall, accurate hand–eye calibration and the human–robot cooperative physical guiding groove likely improved angular control, which may explain the superior angular-control performance of our method.

**Table 3.** Osteotomy error of fibula. Values are reported as means ± SD. Boldface indicates the best performance, i.e., the lowest error value in each column.

Methods	Angle error/(°)	Centroid distance error/mm
CRAR [16]	5.48 ± 2.06	1.86 ± 0.43
ACPM [24]	3.51 ± 2.69	2.40 ± 2.06
AROF [7]	5.04 ± 2.61	**1.03** ± **0.68**
CORPPP [2]	4.11 ± 2.60	1.23 ± 0.98
Clinical (CG [25])	–	1.30 ± 0.59
RAMRS (proposed)	**3.19** ± **1.39**	1.28 ± 0.59

RAMRS, robot-assisted and augmented reality-guided maxillofacial reconstruction system; CG, clinical guideline; CRAR, contour registration-based augmented reality system; ACPM, assisted computer modeling and prototyping method; AROF, AR-guided osteotomy of the fibula method; CORPPP,combined osteotomy and reconstruction pre-shaped plate position

For mandible osteotomy, 2 indicators are used: (a) the Euclidean distance of characteristic points and (b) the volumetric error of the resected segment (the ratio between the absolute difference of planned and actual volumes and the planned volume). Table 4 reports the comparison against public baselines. RAMRS achieves feature–point accuracy that is numerically slightly better than the clinical guide and performs favorably against CRAR and AROF. In addition, RAMRS reports a low volumetric error, whereas the image-guided sagittal saw method shows higher errors, likely due to frequent viewpoint switching between the surgical field and the display during cutting. Note that some baselines do not report volume errors, precluding a comprehensive volumetric comparison across all methods.

**Table 4.** Osteotomy error of mandible. Values are reported as means ± SD. Boldface values indicate the lowest error values among the compared methods.

Methods	Feature point distance error/mm	Volume error/%
IGSS [26]	2.63 ± 1.27	8.55 ± 5.51
CRAR [16]	1.41 ± 0.61	–
AROF [7]	1.62 ± 0.38	–
Clinical (CG [41])	1.20 ± 0.60	–
RAMRS (proposed)	**1.18** ± **0.59**	**4.34** ± **2.76**

RAMRS, robot-assisted and augmented reality-guided maxillofacial reconstruction system; CG, clinical guideline; CRAR, contour registration-based augmented reality system; IGSS, image-guided sagittal saw method

### Experiments on skeletal specimens: Reconstruction

#### Simulated experiments for QR marker registration

Marker registration accuracy highly depends on corner-detection accuracy. Intraoperative corner deviations mainly arise from manual operational perturbations. To validate the effectiveness of the proposed RCC method, we introduce perturbation-noise analysis to evaluate the robustness of RCC optimization under simulated human-induced noise. We therefore build a controlled simulation and compare it against 2 representative methods. The ideal square marker has side length s=50mm, with ground-truth corners ${\left\{Q_{i}\right\}}_{i=1}^{4}$ lying on a common plane. To mimic clinical deviations caused by lever deformation or light contact, we replace fixed-variance noise with a bounded random perturbation of variable strength. For each trial, we draw an amplitude δ∼U(0,3mm) and then add zero-mean uniform noise to each coordinate, $\varepsilon_{i}∼U{(-\delta ,\;\delta )}^{3}$, yielding $P_{i}=Q_{i}+\varepsilon_{i}$. Given the same inputs $\left\{P_{i}\right\}$, we compare 3 methods: (a) least-squares method (LS) [39], which performs a total least-squares plane fitting and projection, then applies constrained Procrustes alignment to a square template to jointly estimate translation, rotation, and scale by minimizing an $L_{2}$ objective; (b) constrained-optimization method (CONS) [40], which formulates rectification and square-prior enforcement as a constrained optimization problem, first seeking an approximately fronto-parallel view via homography and then imposing square-consistency constraints to regularize the geometry; and (c) our proposed method (RCC). All outputs $\left\{{\hat{Q}}_{i}\right\}$ are expressed in the marker-plane coordinate system for evaluation.

To characterize proximity to an ideal marker board and geometric consistency, we employ 4 complementary metrics: (a) Corner displacement, defined as the overall average deviation of the 4 corners from their ground truth measured by the $L_{2}$ norm. This metric reflects overall localization accuracy and tail risk; (b) Area error, the relative error between the area of the estimated quadrilateral and that of the ideal square. This metric indicates whether the global scale is stretched or compressed; (c) Orthogonality deviation, the extent to which the 4 interior angles depart from right angles, reported as the mean deviation across the 4 corners; (d) Opposite-edge parallelism deviation, the directional consistency between the 2 pairs of opposite edges, reported as the average of the 2 pairwise angle deviations. This metric identifies trapezoidal distortion caused by perspective or affine shear. For each trial, all 3 methods are run on the same $\left\{P_{i}\right\}$; the metrics are computed, and the results are reported as mean ± SD over N=100 trials. Quantitative results are summarized in Table 5. Statistical significance was assessed using a Friedman test for overall paired differences across the 3 methods, followed by Bonferroni-corrected Wilcoxon signed-rank tests for post hoc pairwise comparisons.

**Table 5.** Comparison of marker-corner optimization methods. Values are reported as means ± SD. Boldface values indicate the best performance, the lowest error or deviation values among the compared methods. Statistical significance was assessed using the Friedman test followed by Bonferroni-corrected Wilcoxon signed-rank tests. For all 4 metrics, the differences between RCC and LS, as well as RCC and CONS, are highly significant (P<0.001).

Methods	Corner displacement /mm	Area error /%	Ortho deviation /(°)	Parallel deviation /(°)
LS [39]	2.98 ± 0.61	0.95 ± 0.40	3.35 ± 1.20	2.88 ± 1.18
CONS [40]	3.02 ± 0.72	**0.55** ± **0.34**	3.63 ± 1.67	3.33 ± 1.79
RCC	**2.21** ± **0.56**	0.72 ± 0.21	**1.98** ± **0.90**	**1.15** ± **1.02**

RCC, rotating-calipers-based compensation; CONS, constrained-optimization method; LS, least-squares method

RCC achieved the best overall geometric fidelity by jointly modeling a global square prior, local point-geometry constraints, and a robust ordering strategy. Compared with CONS, RCC showed a slightly higher area error. This is likely because CONS more strongly enforces global scale consistency, whereas RCC prioritizes corner stability and orthogonality and therefore tolerates minor scale drift to improve robustness. Plane-consistency constraints mitigated noncoplanarity. Rotating-calipers ordering reduced jitter-induced corner misordering. Square-scale recovery alleviated homography-driven shape overfitting. Together, these mechanisms suppressed distortions caused by noise and ordering errors, preserved right angles and parallelism, and yielded stable performance across repeated trials.

#### Fusion error

Fusion accuracy is a fundamental metric for evaluating AR guidance because it directly reflects the alignment between the virtual model and the real surgical scene. During defective mandibular reconstruction, surgeons rely on visual guidance for segment positioning, making the procedure sensitive to pixel-to-physical scaling and viewing geometry. Therefore, lower fusion error provides more reliable intraoperative guidance. Two feature points are first selected, and the connecting segment between them is adjusted to be as parallel as possible to the camera imaging plane. We then measure their real-world distance ${\mathrm{dis}}_{\text{real}}$ and image pixel distance ${\mathrm{dis}}_{\text{pixel}}$, and compute a pixel-to-metric scale using Eq. (14):

$$\text{Rate}=\frac{{\mathrm{dis}}_{\text{real}}}{{\mathrm{dis}}_{\text{pixel}}}=\frac{\mathrm{dis}\;\left(p_{\text{real}\_1,\;}p_{\text{real}\_2}\right)}{\mathrm{dis}\;\left(p_{\text{pixel}\_1,\;}p_{\text{pixel}\_2}\right)}$$ (14)

**Fig. 7.**
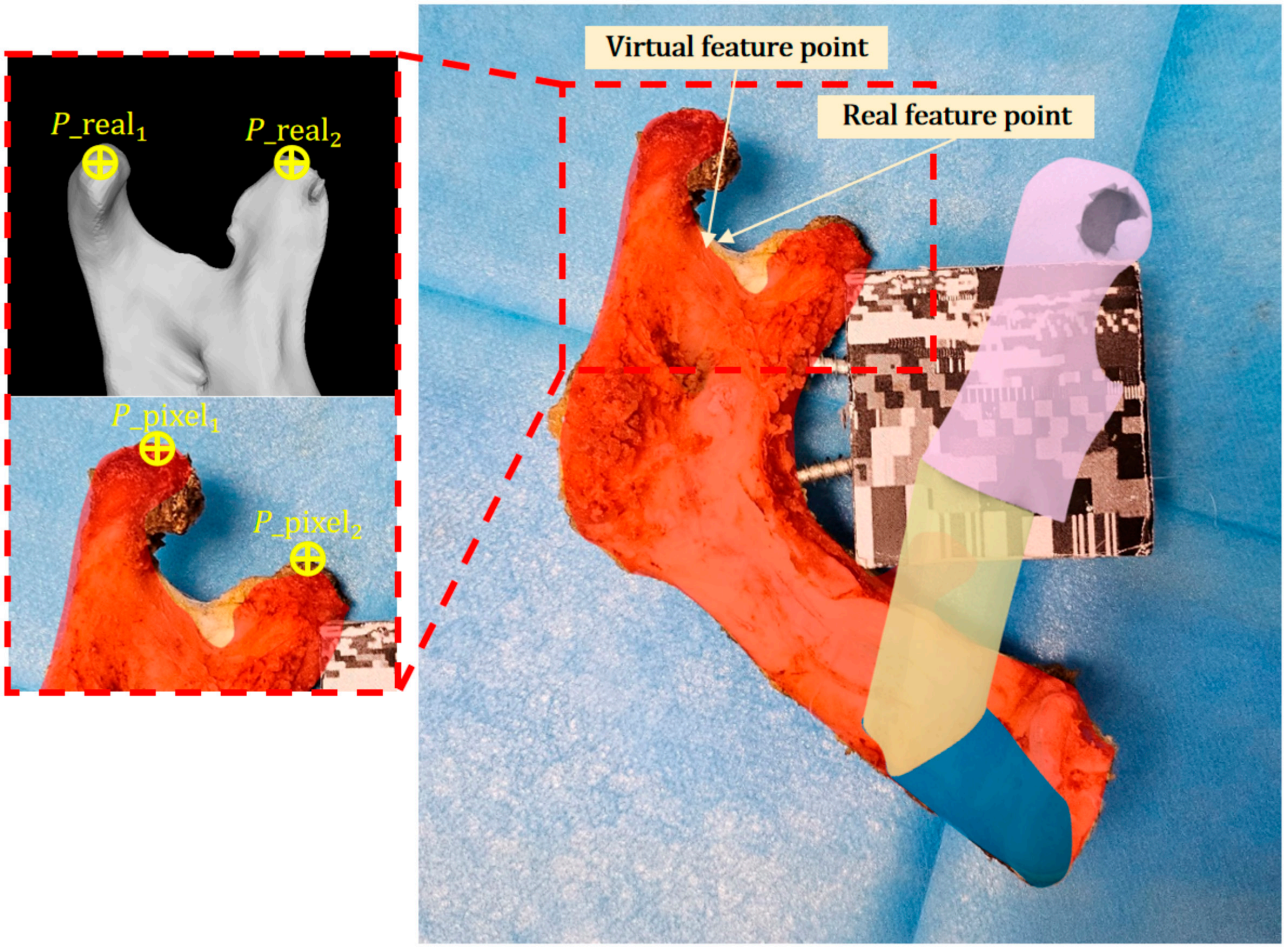
Fusion error diagram: The selection of the pixel distance and the real distance.

For each remaining feature point (see Fig. 7), we measure the pixel distance between its real position in the image and its virtual projection and convert it to metric units via

$${\text{Error}}_{\text{fusion}}=\text{rate}\cdot \mathrm{dis}\;\left(p_{\text{realpixel}},\;p_{\text{virtualpixel}}\right)$$ (15)

where $p_{\text{realpixel}}$ and $p_{\text{virtualpixel}}$ denote the pixel coordinates in the real and virtual images, respectively. At least 5 feature points are selected per defective mandible, and their fusion errors are averaged to obtain a case-level fusion error.

We then compare 3 corner-optimization strategies prior to AR fusion: (a) LS; (b) constrained optimization (CONS); and (c) our proposed method (RCC). For each case, the corners estimated by each method are used to compute the AR projection and the corresponding fusion error as defined above. Results are reported as mean ± SD across cases, and RCC is evaluated against LS and CONS in terms of absolute fusion error as well as relative improvement. Statistical significance was assessed using a Friedman test for overall paired differences, followed by Bonferroni-corrected Wilcoxon signed-rank tests for post hoc pairwise comparisons.

Table 6 indicates that RCC achieves the lowest fusion error with a more concentrated distribution and the smallest worst-case deviation. The *P* values likely reflect a large within-case effect size and high consistency of the fusion error reduction across cases under a paired design, which controls intercase variability and thus enhances statistical power. Mechanistically, imposing plane consistency suppresses out-of-plane jitter and stabilizes the metric frame; rotating-calipers ordering resolves vertex permutations and enforces a convex, consistent sequence; and square-scale recovery anchors the solution to a metric prior, correcting shear and aspect drift without overfitting to a single homography. This combination reduces degrees of freedom, improves numerical conditioning, and limits error propagation from local noise or hand-induced lever deformation, yielding more reliable pixel-to-metric alignment than the 2 baselines.

**Table 6.** Fusion error of the mandible. Boldface values indicate the best performance, the lowest fusion error values among the compared methods. P<0.001 for the overall Friedman test, as well as for all post hoc pairwise comparisons against RCC (Bonferroni-corrected Wilcoxon).

Method	Mean ± SD	Median [IQR]	Max	vs. RCC /%
	/mm	/mm	/mm	improvement
LS [39]	2.19 ± 0.58	2.07 [1.74, 2.49]	3.68	+42.5
CONS [40]	2.43 ± 0.49	2.36 [2.08, 2.73]	3.55	+48.1
RCC	**1.26** ± **0.42**	**1.18** [0.94, 1.47]	**2.31**	—

RCC, rotating-calipers-based compensation; CONS, constrained-optimization method; LS, least-squares method; IQR, interquartile range

#### Reconstruction error

Clinical metrics in maxillofacial reconstruction are primarily used to assess postoperative outcomes. Key assessment parameters include: (a) condylar displacement, i.e., the change in bicondylar span reflecting biomechanical integrity; (b) angular–span deviation, i.e., the change in bilateral mandibular-angle distance affecting spatial symmetry; (c) morphological angulation error at the mandibular angle, which influences facial contour; and (d) volumetric congruence, i.e., the ratio of reconstructed-to-planned bone volumes after alignment as a geometric consistency metric. This multidimensional assessment captures both anatomical precision, quantifying the spatial restoration of functional landmarks, and computational geometry matching, verifying adherence to the surgical plan.

As summarized in Table 7, experimental results showed that RAMRS achieved the lowest mandibular-angle distance and direction errors among the evaluated methods. Compared with the commonly used prebent titanium plate (ST), RAMRS reduced both the bicondylar-span error and the mandibular-angle distance error. For bicondylar span, RAMRS performed slightly worse than CRAR but better than CORPPP and computer-aided design and computer-aided manufacturing, possibly because CRAR provided additional positioning guidance. Overall, the synergy of hand–eye calibration and physical guidance improved angular control while maintaining good positional accuracy.

**Table 7.** Accuracy measurement of reconstruction for the defective mandible. Values are reported as means ± SD. Some referenced studies reported only the mean. Boldface values indicate the lowest error values among the compared methods with available data for each metric.

Methods	Condylar distance error /mm	Mandibular angle distance error/mm	Mandibular angle error/(°)	Fusion distance error /%
CRAR [16]	**0.93** ± **0.63**	2.01 ± 2.49	6.81 ± 2.21	–
CORPPP [2]	1.73 ± 1.13	1.86 ± 0.96	–	–
CAD-CAM [42]	1.60	1.70	–	–
RAARS [38]	–	–	–	14.86 ± 3.21
Clinical (ST [43])	1.85	1.50	–	–
RAMRS (proposed)	1.38 ± 0.32	**1.36** ± **0.48**	**3.62** ± **0.55**	**7.99** ± **1.84**

RAMRS, robot-assisted and augmented reality-guided maxillofacial reconstruction system; CRAR, contour registration-based augmented reality system; CORPPP, combined osteotomy and reconstruction pre-shaped plate position; CAD-CAM, computer-aided design and computer-aided manufacturing; RAARS, robot-assisted augmented reality surgical system

### Experiments on cadaver specimens

**Fig. 8.**
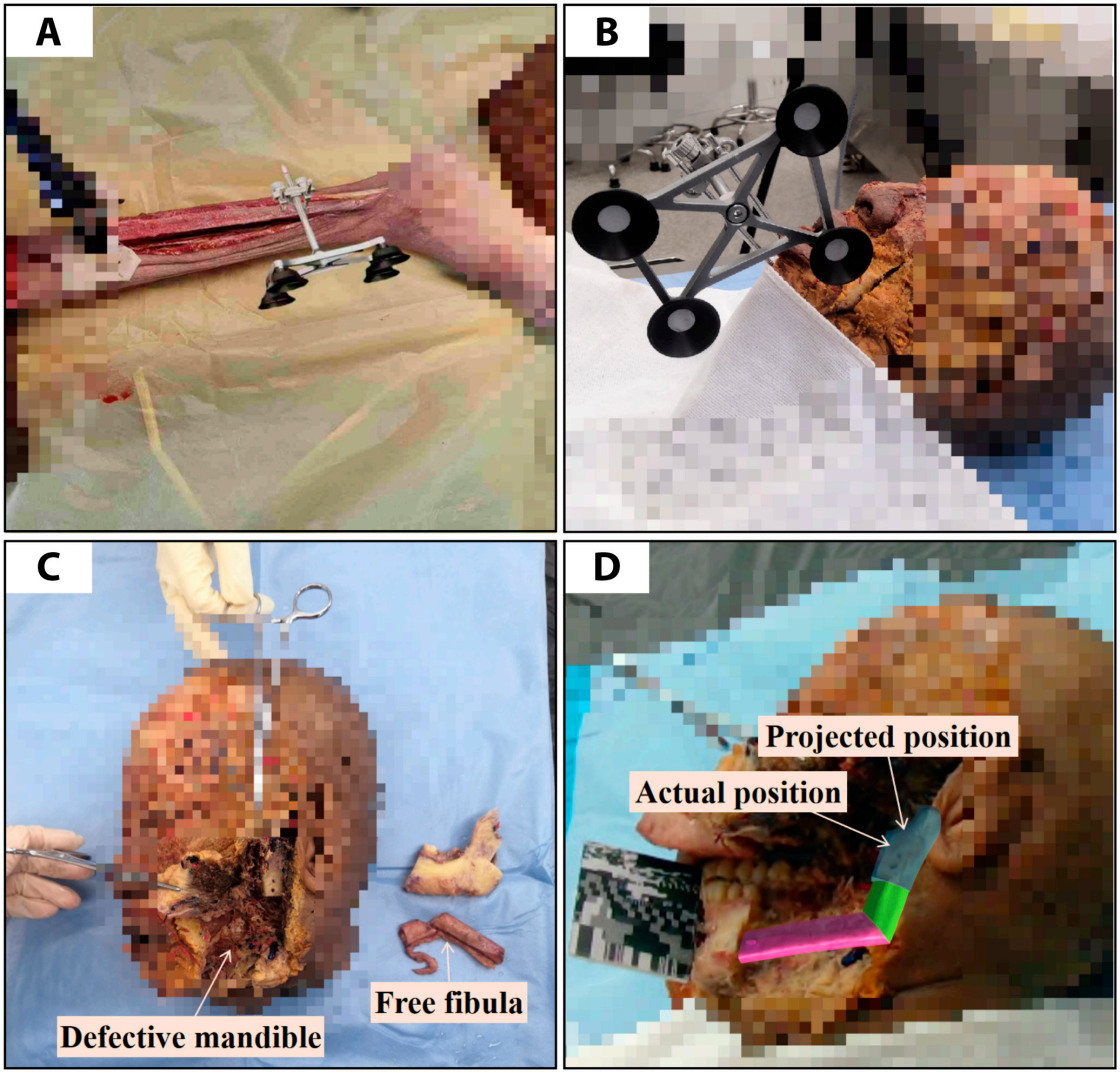
Robot-assisted and augmented reality-guided maxillofacial reconstruction system (RAMRS) for experiments on cadaver specimens. (A) Scene of osteotomies performed on the calf. (B) Scene of osteotomies performed on the head. (C) Fibular segments and defective mandible after osteotomy. (D) Projection scene of the preoperative plan on the defective area.

Compared with ex vivo specimens, cadaveric specimens preserved extraosseous muscles and other soft tissues. Therefore, we performed experiments on cadavers to evaluate the system under surgical conditions that more closely approximated clinical practice. As depicted in Fig. 8A, after the incision of the skin and muscle tissue, a 20-cm fibula was exposed. Subsequently, a tracking marker was inserted, and the fibula was secured with a fixation bracket. To minimize fibula movement during the operation, 2 wooden blocks were placed on either side of the calf. In Fig. 8B, the head was positioned on a table. Following clinical guidelines, skin and muscle tissues were incised along the jaw-labial groove and mandibular line. The optical tracking marker was nailed into the mental prominence of the mandible.

Precise osteotomy was executed based on preoperatively planned volume. Fig. 8C depicts the morphology of the defective mandible and free fibula flap after osteotomy. Subsequently, as shown in Fig. 8D, the preplanned free fibula was projected onto the defective area using the method. Guided by AR, the surgeon positioned the free fibulas before securing them with screws and drilling the titanium plate.

#### Quantitative analysis

Table 8 indicates that the osteotomy error remained within 2 mm. These results confirmed robust localization and execution consistency in RAS mode. Compared with skeletal-specimen tests, cadaver errors were mildly elevated, plausibly due to residual soft tissue around the bone: Incidental contact and compression by the robotic arm during motion can induce small specimen displacements that degrade cutting accuracy. Most prior work remained at the level of models or idealized experimental settings. Systematic validation on ex vivo specimens, and especially on cadavers, was still limited. We validated the system under more clinically relevant conditions, representing an advance over prior work.

**Table 8.** Osteotomy error of the mandible and fibula. Values are reported as means ± SD.

	Mandible		Fibula	
	Feature-point distance error	Volume error	Angle error	Centroid distance error
	/mm	/%	/(°)	/mm
Error	1.72 ± 0.63	6.51 ± 2.83	3.08 ± 0.78	1.47 ± 0.49

Following mandibular osteotomy, the condyle remained adherent to surrounding soft tissue, preventing a reliable bicondylar-span measurement; thus, condylar-distance error was not computed. As summarized in Table 9, reconstruction errors for the cadaveric mandible are small. The errors are modestly higher than in skeletal-specimen tests, which is consistent with the limited exposure and reduced postresection bony surface available for registration in cadaver procedures aimed at minimizing soft-tissue trauma. In the cadaver study, after muscle dissection at the lower leg and head, RAMRS achieved the osteotomy in 12 min and completed reconstruction in 16 min.

**Table 9.** Reconstruction error in the defective mandible. Values are reported as means ± SD.

	Mandibular angle distance error /mm	Mandibular angle error/(°)	Fusion distance error /%
Error	1.49 ± 0.37	4.83 ± 0.54	6.43 ± 0.84

## Discussion and Conclusion

This study designed and evaluated a robot-assisted and AR-integrated maxillofacial reconstruction system named RAMRS. The goal was to improve osteotomy accuracy and free fibula graft positioning accuracy and to provide coordinated support for osteotomy and reconstruction within a unified workflow. During system development, the platform was codesigned with the surgical team and integrated into the actual intraoperative workflow. Automated calibration and an intuitive human–machine interface were incorporated to improve operability and usability. The system also provided visual navigation and real-time feedback to support spatial orientation, which helped surgeons perform intraoperative procedures more stably and precisely. In addition, this study proposed a calibration compensation framework based on rotating calipers. This framework was used to improve QR marker fusion accuracy in marker-based tracking, thereby enhancing the visual alignment quality of AR guidance during reconstruction.

Compared with existing studies on maxillofacial reconstruction, the main contribution of this work is the construction and validation of a unified workflow that covers the key steps of osteotomy, positioning, and fixation. Previous studies have often focused on a single step, with limited attention to cross-step coordination and overall error propagation. Accordingly, this study quantitatively evaluated system accuracy and reliability within the proposed framework, including 3D geometric accuracy for osteotomy and reconstruction, as well as 2D fusion error for AR-guided visualization. Validation was first performed on ex vivo bony specimens and then extended to cadaver specimens to assess system performance under conditions that more closely resembled real tissue conditions. However, the comparative analysis in this paper is mainly based on reported metrics from published studies and should be regarded as a literature-level reference. In addition, differences in hardware platforms and specimen conditions exist across studies. Therefore, we consider that RAMRS shows competitive performance on comparable metrics and demonstrates potential for further translational validation.

Although the proposed system has been validated on ex vivo and cadaveric specimens, real clinical use involves more complex intraoperative disturbances. These include fluid contamination and line-of-sight occlusion. Both may compromise the stability of marker detection. Under the current system configuration, the optical tracking module is expected to maintain basic functionality under limited fluid interference, provided that the geometric features of the markers remain visible. However, persistent blood coverage or repeated saline splashing may still reduce tracking stability. By contrast, the QR code-based AR module is likely to be more sensitive to contamination, because its performance depends more directly on the quality of the 2-dimensional visual input captured by the camera. Blood contamination, soft tissue interference, or partial marker occlusion may therefore reduce recognition accuracy and impair fusion performance. In actual surgery, transient contamination may cause temporary fluctuations in visualization and tracking. Persistent contamination may require active cleaning of the markers or adjustment of the viewing angle to restore performance. At present, the system reduces occlusion risk through optimized device layout, marker placement, and intraoperative line-of-sight management. Routine irrigation and wiping are also used to mitigate contamination effects on tracking and visualization. However, system robustness under sustained occlusion and persistent contamination has not yet been systematically validated. In addition, the calibration compensation method, which is a key component of the ARR module, still requires further evaluation under extreme noise conditions.

Future work will be directed by practical clinical needs. We will first perform systematic robustness assessments in high-risk scenarios and integrate more robust image enhancement and marker detection methods to enhance visualization stability and registration reliability under diverse intraoperative conditions. We will then further explore vision-based vessel segmentation and distance warning mechanisms to mitigate the risk of vascular injury during osteotomy. Meanwhile, efforts in regulatory preparation, workflow integration, and standardized operational research will continue in order to support future prospective clinical evaluation.

## Data Availability

Data to support the findings of this study are available from the first author upon request.
